# New evidence on the impacts of cross‐market hospital mergers on commercial prices and measures of quality

**DOI:** 10.1111/1475-6773.14291

**Published:** 2024-04-23

**Authors:** Daniel R. Arnold, Jaime S. King, Brent D. Fulton, Alexandra D. Montague, Katherine L. Gudiksen, Thomas L. Greaney, Richard M. Scheffler

**Affiliations:** ^1^ School of Public Health University of California, Berkeley Berkeley California USA; ^2^ Faculty of Law The University of Auckland Auckland New Zealand; ^3^ University of California College of the Law, San Francisco San Francisco California USA; ^4^ School of Public Health and Goldman School of Public Policy University of California, Berkeley Berkeley California USA

**Keywords:** cross‐market, health care competition, hospitals, price, quality, serial acquisitions

## Abstract

**Objective:**

To examine the impact of “cross‐market” hospital mergers on prices and quality and the extent to which serial acquisitions contribute to any measured effects.

**Data Sources:**

2009–2017 commercial claims from the Health Care Cost Institute (HCCI) and quality measures from Hospital Compare.

**Study Design:**

Event study models in which the treated group consisted of hospitals that acquired hospitals further than 50 miles, and the control group was hospitals that were not part of any merger activity (as a target or acquirer) during the study period.

**Data Extraction Methods:**

We extracted data for 214 treated hospitals and 955 control hospitals.

**Principal Findings:**

Six years after acquisition, cross‐market hospital mergers had increased acquirer prices by 12.9% (CI: 0.6%–26.6%) relative to control hospitals, but had no discernible impact on mortality and readmission rates for heart failure, heart attacks and pneumonia.

For serial acquirers, the price effect increased to 16.3% (CI: 4.8%–29.1%). For all acquisitions, the price effect was 21.8% (CI: 4.6%–41.7%) when the target's market share was greater than the acquirer's market share versus 9.7% (CI: −0.5% to 20.9%) when the opposite was true. The magnitude of the price effect was similar for out‐of‐state and in‐state cross‐market mergers.

**Conclusions:**

Additional evidence on the price and quality effects of cross‐market mergers is needed at a time when over half of recent hospital mergers have been cross‐market. To date, no hospital mergers have been challenged by the Federal Trade Commission on cross‐market grounds. Our study is the third to find a positive price effect associated with cross‐market mergers and the first to show no quality effect and how serial acquisitions contribute to the price effect. More research is needed to identify the mechanism behind the price effects we observe and analyze price effect heterogeneity.

**What is known on this topic**

- Over half of the hospital mergers in the last decade have been cross‐market.
- Cross‐market hospital mergers lead to higher hospital prices.

**What this study adds**

- Serial acquirers are significant contributors to estimated cross‐market price effects.
- We find no discernible impact of cross‐market mergers on mortality and readmission rates for heart failure, heart attacks and pneumonia.
- Overall, this study provides further evidence that cross‐market hospital mergers lead to price increases and novel findings of no quality effect and the impact of serial acquirers on the price effect. More antitrust scrutiny of these mergers—particularly those of serial acquirers—appears prudent given the current state of highly concentrated hospital markets in the United States.

## 1 INTRODUCTION

U.S. hospitals have been consolidating for decades. Between 1998 and 2017 there were 1577 hospital mergers with 456 occurring from 2013 to 2017.^1^ By 2016, 90% of Metropolitan Statistical Areas (MSAs) were highly concentrated according to the U.S. Department of Justice (DOJ) and Federal Trade Commission (FTC)'s Horizontal Merger Guidelines.^2^ Hospitals joining systems is a primary driver of this increase in concentration. From 1970 to 2019, the percentage of hospitals in multi‐hospital systems increased substantially from 10% to 67%.^3^

As hospital systems have expanded, they've extended into regions where they previously had no presence.^4^ A recent study found 55% of the 1500 hospitals targeted for a merger or acquisition from 2009 to 2019 operated in a commuting zone that the acquirer did not previously operate in.^3^ The price and quality effects of these “cross‐market” hospital mergers and acquisitions (M&A) are the focus of this paper.

Two previous empirical studies examine the price impacts of cross‐market hospital mergers – Lewis and Plum (2017) and Dafny, Ho, and Lee (2019).^5^, ^6^ Lewis and Pflum (2017) found that prices at target hospitals involved in cross‐market mergers increased by about 17% more than unacquired, stand‐alone hospitals, with these increases reaching 29% for targets acquired by large systems and 33% for small targets being acquired. The authors additionally showed that out‐of‐market mergers lead to a relaxation of competition; that is, the prices of nearby competitors to acquired hospitals increase by around 8%.^5^

Dafny, Ho, and Lee (2019) found that hospitals involved in cross‐market mergers had price increases of 7% to 10% relative to control hospitals if the acquisition was in‐state, but did not find relative price increases when the acquisition was out‐of‐state. The price effect persisted when the target hospitals were excluded from the model, meaning the acquiring system's hospitals also had relative price increases. The price increase of the acquiring system's hospitals climbed to 31% when the acquirer had a below‐median market share and the target had an above‐median market share, and the price increase was 18% in the opposite situation, when the acquirer had an above‐median market share and the target had a below‐median market share.^6^

The contribution of our paper is threefold. First, we add to the empirical evidence of the price effects of cross‐market hospital mergers by providing the first evidence using the actual prices paid by commercial insurers (and consumers through out‐of‐pocket payments). The two previous empirical papers on the price effects of cross‐market mergers calculated prices by adjusting revenue data collected at the hospital level. Second, we provide the first evidence of the quality effects of cross‐market mergers. Compared with the empirical evidence on the price and quality effects of horizontal hospital mergers, the empirical evidence on the effects of cross‐market hospital mergers is sparse.

Finally, we are the first to present evidence of the price effects generated by serial cross‐market acquirers. We do this by utilizing a new difference‐in‐differences estimator that allows treated units to receive multiple changes in their treatment dose by redefining the “event” as the first time a group's treatment changes.^7^ Accounting for increases in treatment dose is particularly important in our setting as it was very common for the acquiring systems in our sample to acquire a cross‐market hospital in more than 1 year during our study period. Importantly, this allows our work to complement Dafny, Ho, and Lee (2019), which limited its treatment sample to hospitals experiencing a treatment only once during the five‐year period spanning the transaction generating that treatment. The authors noted that this means the transactions included in their final analysis sample “involve smaller acquirers (as measured by the number of facilities), as larger acquirers tend to engage in multiple closely timed acquisitions.”^6^ The new estimator allows us to estimate the impact of cross‐market mergers on the prices of hospitals that are part of the large systems that serially acquire cross‐market hospitals.^7^

We focus on the cross‐market price effect at acquiring hospitals as opposed to target hospitals. Lewis and Pflum (2017) convincingly show that cross‐market mergers lead to higher prices at target hospitals.^5^ But from an antitrust perspective, challenging cross‐market mergers is less of an uphill battle if the evidence is clear that cross‐market mergers allow acquirers to increase their prices, because prices at the acquirer are not likely to increase due to a “change in control” or better quality. Change in control theory in the context of cross‐market mergers boils down to the acquirer being able to increase prices at the target because the target wasn't maximizing profit; either because it was nonprofit and maximizing profit wasn't its objective, or because it didn't have the bargaining skill to negotiate high prices.^8^ Acquirers by definition do not experience a change of control and thus this explanation for higher prices after a cross‐market merger is ruled out. It also seems unlikely that an increase in quality could explain acquirer price increases after a cross‐market merger. Acquirers are often large health systems whereas targets are frequently independent hospitals.^3^ It seems unlikely that a large health system's quality would improve by merging with an independent hospital. However, despite acquirer quality improvements being a priori unlikely, we test this empirically to confirm our intuition.

### 1.1 Potential mechanisms

To date economists have proposed five mechanisms for cross‐market price increases: (1) common customers, (2) tying, (3) change in control, (4) hospital quality improvements, and (5) multimarket contact. As noted in the previous paragraph, our focus on acquirer prices is meant to make it unlikely that (3) and (4) are the mechanisms driving our result. We discuss (1), (2), and (5) briefly for the remainder of this section (see King et al. 2023 for a more detailed review of these mechanisms).^8^

The common customer theory states that cross‐market price increases can arise from the market linkages created by the existence of a common customer. The common customer could be an employer or insurer. Employers (or the insurers who sell to them) need provider networks that span multiple patient markets if they have employees in multiple markets. For instance, a large national employer like Wal‐Mart needs a health plan that has provider networks in all parts of the country. Wal‐Mart could contract with a different local health plan in all parts of the U.S., but it's easy to see how contracting with one insurer that has created a provider network that covers the whole country could be desirable.

Tying deals with how a firm with market power in one market (the tying market) can tie its sales in that market with its sales in a second market (the tied market). Tying by a monopolist can reduce the sales of its competitors in the tied market and lower their profits below a level that would justify continuing operations.^9^ Bundling across markets can also increase the bargaining strength of firms and lead to higher prices without disadvantaging rivals.^10^, ^11^

Multimarket contact is the notion that as hospital systems grow they will increasingly come into contact with each other in more and more markets throughout the U.S. Bernheim and Whinston^12^ show how multimarket contact can lead to collusive behavior. For example, if systems A and B know they are going to compete against each other several times for inclusion in insurers' networks, it may make sense for them to not compete as much on price as they would have in a one‐off situation for fear of retaliation.^13^, ^14^

## 2 DATA

### 2.1 Hospital prices

We utilized 2009–2017 commercial claims from the Health Care Cost Institute (HCCI)'s 1.0 database to construct our measure of hospital price. HCCI 1.0 pools medical claims data from three large U.S. health insurers—Aetna, Humana, and UnitedHealth. The HCCI data covers on average 45 million under age 65 individuals with commercial insurance per year from 2009 to 2017 and includes observations from every U.S. state and metropolitan statistical area. Our price measure is the amount paid to a hospital for a standardized inpatient admission. The amount paid is the amount paid by the health insurer plus the out‐of‐pocket amount paid by the patient, including deductibles, copayments, and coinsurance. We standardized prices by dividing the total amount paid for admissions to a hospital by the number of standardized admissions. A standardized admission is an admission of average intensity, with a relative weight equal to one, but admissions that deviate from the average intensity receive a relative weight that reflects their intensity. We used MS‐DRG relative weights, which assign relative weights based on the clinical characteristics of the inpatient stay and the expected resource requirements. For example, a kidney transplant is more complicated and requires more clinical resources than an uncomplicated childbirth. In 2017, a kidney transplant had a relative weight of 3.2, and, therefore, accounted for 3.2 standardized admissions, whereas an uncomplicated childbirth, which had a relative weight of 0.6, accounted for 0.6 standardized admissions. This data has been used in several studies that have analyzed the impact of health care consolidation on prices, but has never been used in the context of cross‐market hospital mergers.^15^, ^16^, ^17^

### 2.2 Hospital quality

Our measures of hospital quality were extracted from CMS' Hospital Compare. We extracted six measures of quality for which data was consistently reported during from 2009 to 2017: 30‐day mortality and readmissions rates for heart failure, heart attacks, and pneumonia. All six measures can range from 0 to 100 with lower values indicating better quality. This data has been used in several studies to analyze the impact of hospital consolidation on quality.^16^, ^18^

### 2.3 Cross‐market hospital mergers

We began by constructing a panel of the short‐term community hospitals using Fiscal Years 2009–2017 of the American Hospital Association (AHA)'s Annual Survey. We then used hospital ownership information from AHA to determine whether a hospital was involved in M&A activity during a given year. We identified hospitals that were M&A targets as those whose system identifiers changed between years in the AHA data. We identified acquirers as hospitals in systems containing hospital targets, but whose system identifiers did not change. In the case when a merger led to all hospitals in the merged system obtaining a new system identifier, we categorized the hospitals in the system that had more hospitals pre‐merger as acquirer hospitals and the hospitals in the system with fewer hospitals pre‐merger as target hospitals.

### 2.4 Control variables

We included a set of time‐varying hospital‐ and county‐level control variables in our models. The hospital‐level control variables were extracted from AHA and included a hospital's number of beds, indicator variables for the hospital's for‐profit, government, or teaching hospital status, and the hospital's share of inpatient days from Medicare and Medicaid enrollees (to control for potential cost‐shifting) as well as its number of technologies. The county‐level control variables included number of hospitals, uninsured rate, median household income, population, and unemployment rate.

## 3 EMPIRICAL STRATEGY

We used the event study estimator developed by de Chaisemartin and D'Haultfoeuille (Forthcoming) (hereafter, dCDH estimator) to quantify the impact of cross‐market hospital mergers on the price and quality of acquiring hospitals.^7^ To the best of our knowledge, the dCDH estimator is the first estimator that both (1) incorporates the recent developments in the difference‐in‐differences event study literature^19^ (e.g., accounting for staggered interventions with heterogeneous treatment effects) and (2) enables an estimate of multiple treatments, which is critical for our serial acquisition analysis. The estimator allows treated units to receive multiple changes in their treatment dose by redefining the “event” as the first time a group's treatment changes. Accounting for increases in treatment dose is particularly important in our setting as it was very common for the acquiring systems in our sample to acquire a cross‐market hospital in more than 1 year between 2011 and 2017. Of the 214 acquiring hospitals that met our treatment requirements, only 32 of them acquired a cross‐market hospital in only 1 year from 2011 to 2017. Among the remaining 182 treated hospitals, 96 hospitals were part of systems that acquired a cross‐market hospital in four or more years from 2011 to 2017. These 96 hospitals were spread across 12 systems (see Table A1 in the Supporting Information for the full distribution).

Before presenting the regression model we estimated, we first detail how we constructed our sample of treated and control hospitals. Treated hospitals met the following criteria: (1) they, independently or as part of a system, acquired a hospital (or system) that was further than 50 miles away between 2011 and 2017, with the first acquisition occurring from 2011 to 2015; and (2) they were never a target of an acquisition from 2009 to 2017. The 50‐mile requirement was to ensure that the mergers were safely “cross‐market.” While treated hospitals needed to be more than 50 miles from any target hospital, it could be the case that other hospitals in the acquiring system were within 50 miles. For instance, if a two‐hospital system (hospitals A1 and A2) acquired independent hospital B, and A1 was 100 miles from B and A2 was 25 miles from B, we would consider just A1 to be treated. Other studies use similar distance cutoffs for defining cross‐market. Lewis and Pflum (2017) used 45 miles and Dafny, Ho, and Lee (2019) used 30 min' drive.

The requirement that the first acquisition needed to occur during 2011–2015 means the treated hospitals did not participate in a merger or acquisition transaction for at least 2 years prior to treatment, providing a “clean” pre‐treatment period to assess relative difference‐in‐differences in prices between the treatment and control hospitals prior to the treated period. This requirement also ensures at least 3 years of price data post‐acquisition (including the acquisition year) was available for treated hospitals. Hospitals that were not involved in M&A (either as targets or acquirers) from 2009 to 2017 served as our control hospitals.

The idea behind the dCDH estimator is to take the perspective of a social planner seeking to conduct a cost–benefit analysis comparing hospitals' actual treatments (i.e., acquiring a cross‐market hospital) to the counterfactual “status‐quo” scenario where every hospital would have kept the same treatment as in period 1 (i.e., no cross‐market acquisitions). In our context, the planner wants to know if the cross‐market mergers that took place over the entire duration of the study period led prices and quality to be higher or lower. This means we can account for a common scenario in our data of a treated hospital receiving multiple “doses” in the form of acquiring multiple cross‐market hospitals over our study period. For instance, if a hospital acquired a cross‐market hospital in 2011, 2015, and 2017 we would consider it to have been treated three times. See Supporting Information A for the technical details and identifying assumptions of the dCDH estimator in our context.

## 4 RESULTS

Table 1 shows descriptive statistics for the 214 treated hospitals and 955 control hospitals in our sample. Treated hospitals were more likely to be for‐profit and have a higher share of Medicare inpatient days than control hospitals. They also had fewer beds, a lower share of Medicaid inpatient days, and were less likely to be government or teaching hospitals than control hospitals. In terms of county characteristics, treated hospitals were in counties with a lower population, lower income, and fewer hospitals than control hospitals. They were also more likely to be in the South and West Census Regions than control hospitals.

**TABLE 1:** Attributes of treated and control hospitals.

	Treated hospitals mean (SD)	Control hospitals mean (SD)	*p*‐Value differences in means
Dependent price variable			
ln(Price)	9.35 (0.46)	9.17 (0.54)	<0.01
Price ($)	12,661 (5552)	11,079 (6344)	<0.01
Hospital characteristics			
Beds	187 (192)	206 (216)	<0.01
For‐Profit	0.21 (0.41)	0.02 (0.15)	<0.01
Government	0.06 (0.23)	0.34 (0.47)	<0.01
Teaching	0.05 (0.22)	0.09 (0.29)	<0.01
Medicare Share of IP Days	0.53 (0.14)	0.50 (0.17)	<0.01
Medicaid Share of IP Days	0.19 (0.11)	0.21 (0.15)	<0.01
Technologies	50 (31)	49 (33)	0.65
County characteristics			
Population	486,317 (1,303,104)	713,857 (1,637,878)	<0.01
Median Income ($)	49,863 (12,301)	51,986 (13,923)	<0.01
Uninsured	0.15 (0.06)	0.15 (0.06)	0.02
Unemployed	0.07 (0.03)	0.07 (0.03)	0.13
Rural	0.31 (0.26)	0.31 (0.28)	0.85
Hospitals	4.3 (9.2)	5.8 (11.9)	<0.01
Census region			
Northeast	0.06 (0.23)	0.15 (0.36)	<0.01
Midwest	0.24 (0.43)	0.26 (0.44)	
South	0.41 (0.49)	0.35 (0.48)	
West	0.29 (0.46)	0.24 (0.43)	
Observations	1926	8595	
Unique Hospitals	214	955	

*Note*: Statistics in the table are pooled across years. Treatment hospitals included hospitals (or hospitals within systems) that met the following criteria: (1) hospitals that made an acquisition from 2009 to 2017 of a hospital (or system) that was further than 50 miles away, with the first acquisition occurring from 2011 to 2015; and (2) hospitals that were never a target of an acquisition from 2009 to 2017. Control hospitals were never part of merger activity (either as a target or acquirer) from 2009 to 2017.

Abbreviations: IP, inpatient; ln, natural log; SD, standard deviation.

Figure A1 in the Supporting Information shows the raw price trends for treated and control hospitals. As a reminder, our group of treated hospitals was constructed so that they were first treated during the 2011–2015 time period. The breakdown by treatment year for the 214 treated hospitals in our sample is 80 in 2011, 31 in 2012, 49 in 2013, 37 in 2014, and 17 in 2015. The average price at treated hospitals started higher than that of control hospitals and remained higher throughout our 2009–2017 study period. The average price for treated hospitals grew by 40% over the period (from $10,479 in 2009 to $14,640 in 2017) whereas the average price for control hospitals grew by 39% over the period (from $9184 in 2009 to $12,758 in 2017).

Figure A2 in the Supporting Information splits the treated group of hospitals by whether the hospital was part of a system that acquired cross‐market hospitals in four or more years from 2011 to 2017. The control hospital price trend lines in Figure A2 are the same as the control price line shown in Figure A1. Panel A shows the average price of the 118 treated hospitals whose systems acquired cross‐market hospitals in three or fewer years from 2011 to 2017 grew by 33% (from $11,299 to $15,059). Panel B shows the average price of the 96 treated hospitals whose systems acquired cross‐market hospitals in four or more years from 2011 to 2017 grew by 49% (from $9471 to $14,125).

**FIGURE 1:**
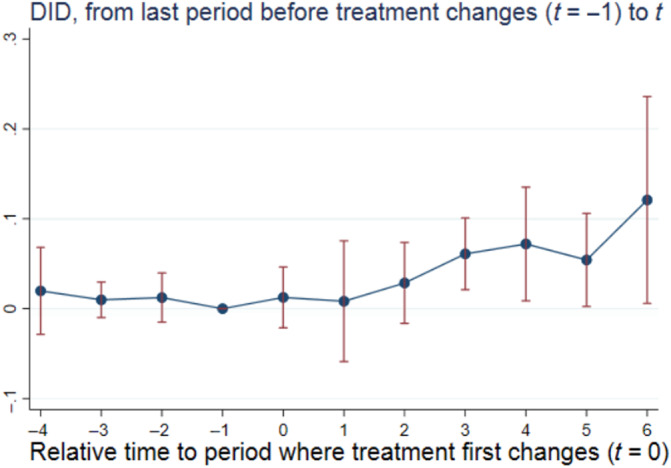
Effect of cross‐market M&A on acquirers' prices. Standard errors were estimated using 100 bootstrap replications clustered at the hospital level. The regression underlying this figure included hospital and year fixed effects as well as time‐varying hospital‐ and county‐level control variables. The hospital‐level control variables included number of beds, indicator variables for the hospital's for‐profit, government, or teaching hospital status, and the hospital's share of inpatient days from Medicare and Medicaid enrollees as well as its number of technologies. The county‐level control variables included number of hospitals, uninsured rate, median household income, population, and unemployment rate. The coefficient estimates corresponding to this figure are available in Table A2 of the Supporting Information. DID, difference‐in‐differences; M&A, mergers and acquisitions; *t*, time since treatment first changes.

Figure 1 graphically depicts the results of our regression analysis (see Table A2 in the Supporting Information for the regression coefficients underlying the figure). The placebo estimates $\left(t=-4\ldots -2\right)$ all hover around zero and are not statistically significant. The $\mathrm{DI}D_{t}$ estimates start out around zero and begin trending up at t=2. By t=3 the coefficient estimate is 0.061 and is statistically significant (p=0.003). By t=6 the coefficient estimate is 0.121 (p=0.039) indicating that prices at hospitals treated for the first time six periods ago are 12.9% (=(exp(0.121)‐1)*100) higher relative to prices at control hospitals.

**FIGURE 2:**
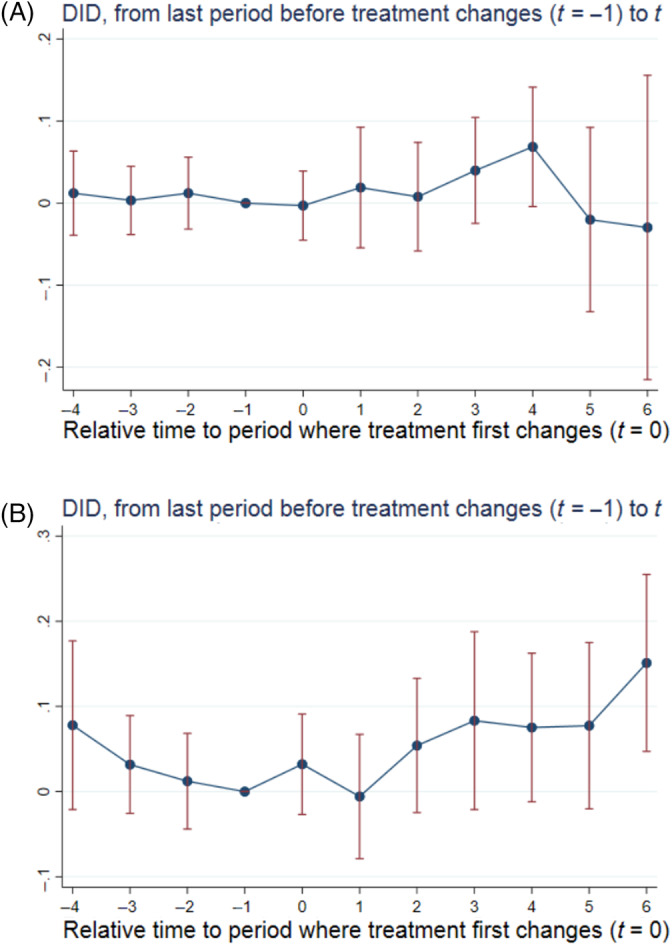
Effect of cross‐market M&A on acquirers' prices by number of years as a cross‐market acquirer. (A) Acquirers in 3 or fewer years. (B) Acquirers in 4 or more years. Standard errors were estimated using 100 bootstrap replications clustered at the hospital level. Panel A includes the 118 treated hospitals that were part of systems that acquired hospitals more than 50 miles away in three or fewer separate years between 2011 and 2017. Panel A includes the 96 treated hospitals that were part of systems that acquired hospitals more than 50 miles away in four or more separate years between 2011 and 2017. The regressions underlying these event study plots included hospital and year fixed effects as well as time‐varying hospital‐ and county‐level control variables. The hospital‐level control variables included number of beds, indicator variables for the hospital's for‐profit, government, or teaching hospital status, and the hospital's share of inpatient days from Medicare and Medicaid enrollees as well as its number of technologies. The county‐level control variables included number of hospitals, uninsured rate, median household income, population, and unemployment rate. DID, difference‐in‐differences; M&A, mergers and acquisitions; *t*, time since treatment first changes.

In Figure 2 we attempt to disentangle the 12.9% price effect. Panel A shows the event study where we keep the control hospitals the same, but the treated group is now the 118 treated hospitals whose systems acquired cross‐market hospitals in three or fewer years from 2011 to 2017. Panel B shows the event study where the control hospitals are the same, but the treated group is the 96 treated hospitals whose systems acquired cross‐market hospitals in four or more years from 2011 to 2017. Panel A again shows no sign of a pre‐trend and the t=4 coefficient of 0.069 is statistically significant (p=0.065), indicating there is still a price effect when the cross‐market acquisition isn't part of an extended string of cross‐market acquisitions in successive years. However, the price effect appears more transitory in this case as the coefficient estimates are directionally negative and not statistically significant in t=5,6.

Panel B, on the other hand, shows a steady and persistent price effect. The t=4 coefficient is 0.075 (p=0.096), and by the time t=6 comes around the coefficient is 0.151 (p=0.004), indicating prices are 16.3% (CI: 4.8%–29.1%) higher at treated hospitals that are part of systems serially acquiring cross‐market hospitals relative to prices at control hospitals.

**FIGURE 3:**
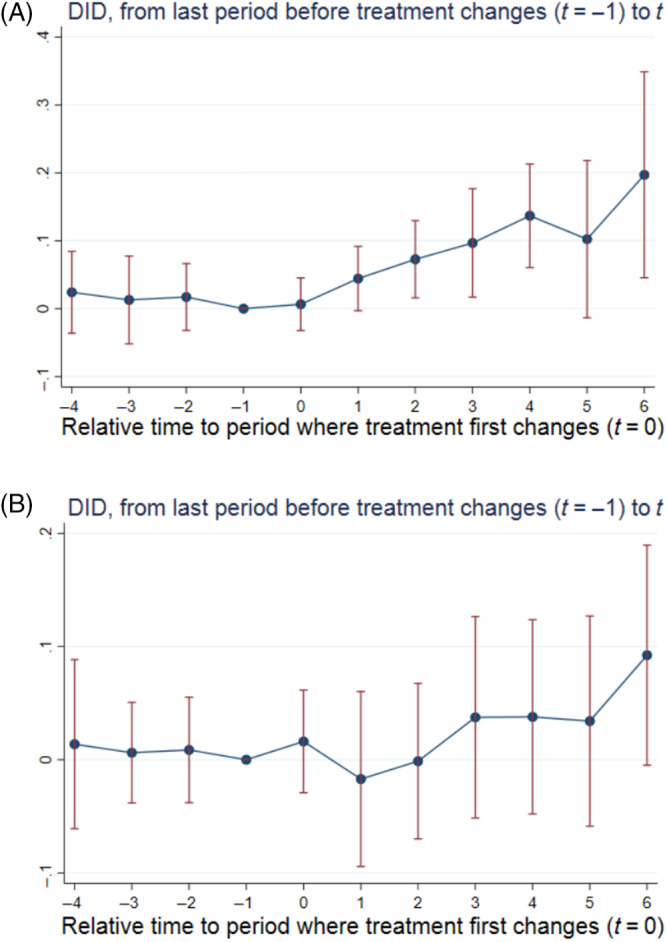
Effect of cross‐market M&A on acquirers' prices by whether the target or acquiring hospital had greater market share. (A) Target Market Share > Acquirer Market Share. (B) Acquirer Market Share > Target Market Share. Standard errors were estimated using 100 bootstrap replications clustered at the hospital level. Panel A includes the 87 treated hospitals whose market shares were lower than those of the first cross‐market targets they acquired during the study period. Panel B includes the 127 treated hospitals whose market shares were higher than those of the first cross‐market targets they acquired during the study period. The regressions underlying these event study plots included hospital and year fixed effects as well as time‐varying hospital‐ and county‐level control variables. The hospital‐level control variables included number of beds, indicator variables for the hospital's for‐profit, government, or teaching hospital status, and the hospital's share of inpatient days from Medicare and Medicaid enrollees as well as its number of technologies. The county‐level control variables included number of hospitals, uninsured rate, median household income, population, and unemployment rate. DID, difference‐in‐differences; M&A, mergers and acquisitions; *t*, time since treatment first changes.

In Figure 3 we show how the price effect differs by whether the acquiring hospital had a higher or lower market share than the target system. Each hospital's market share was measured as its share of admissions among general acute care hospitals located in its county. Next, we compared each treated hospital's market share to the market share of the target it was acquiring. If the target was more than one hospital we calculated the target's market share as the weighted average (using admissions) of the county market shares of its system members. Panel A includes treated hospitals whose market shares were below the market shares of the first cross‐market targets they acquired during the study period. Panel B shows the opposite situation – it includes treated hospitals whose market shares were above the market shares of the first cross‐market targets they acquired during the study period. The average market shares of the targets and acquirers in Panel A were 76% and 56%, respectively. The average market shares of the targets and acquirers in Panel B were 24% and 59%, respectively. Comparing the two event studies plots indicates that the price effect is twice as large when the target's market share is greater than the acquirer's (the t=6 coefficient is a positive and statistically significant $0.197\;\left(p=0.011\right)$ indicating prices 21.8% (CI: 4.6%–41.7%) higher than those at control hospitals, see Panel A) than it is in the reverse situation (the t=6 coefficient is a positive and statistically significant $0.092\;\left(p=0.063\right)$ indicating prices 9.7% (CI: −0.5% to 20.9%) higher than those at control hospitals, see Panel B).

Figure A3 in the Supporting Information delves deeper into the acquirer price effect of cross‐market mergers by assessing whether there is a difference between the price effect of cross‐market mergers that occur within a state and those that cross‐state lines. Among the 214 treated hospitals in our sample, 68 hospitals only experienced out‐of‐state cross‐market mergers during our study period. Our sample also included 60 hospitals that only experienced within state cross‐market mergers during our study period. The remaining 86 treated hospitals experienced some combination of out‐of‐state and within state cross‐market mergers during our study period.

Panel A in Figure A3 shows the event study after removing all treated hospitals in the sample except the 68 hospitals that only experienced out‐of‐state cross‐market mergers during our study period. The figure is very similar to that shown for the full sample, indicating that the price effect for out‐of‐state cross‐market mergers is no different to than it is for other types of cross‐market mergers. Specifically, the *t* = 6 coefficient is identical magnitude to that of the *t* = 6 coefficient in full sample version (0.121 and 0.121), so they are not statistically different. Panel B repeats the analysis using the 60 hospitals that only experienced in‐state cross‐market mergers as the treated hospitals. It's *t* = 6 coefficient (0.130) is not statistically different than the *t* = 6 coefficients in the full sample and Panel A.

**FIGURE 4:**
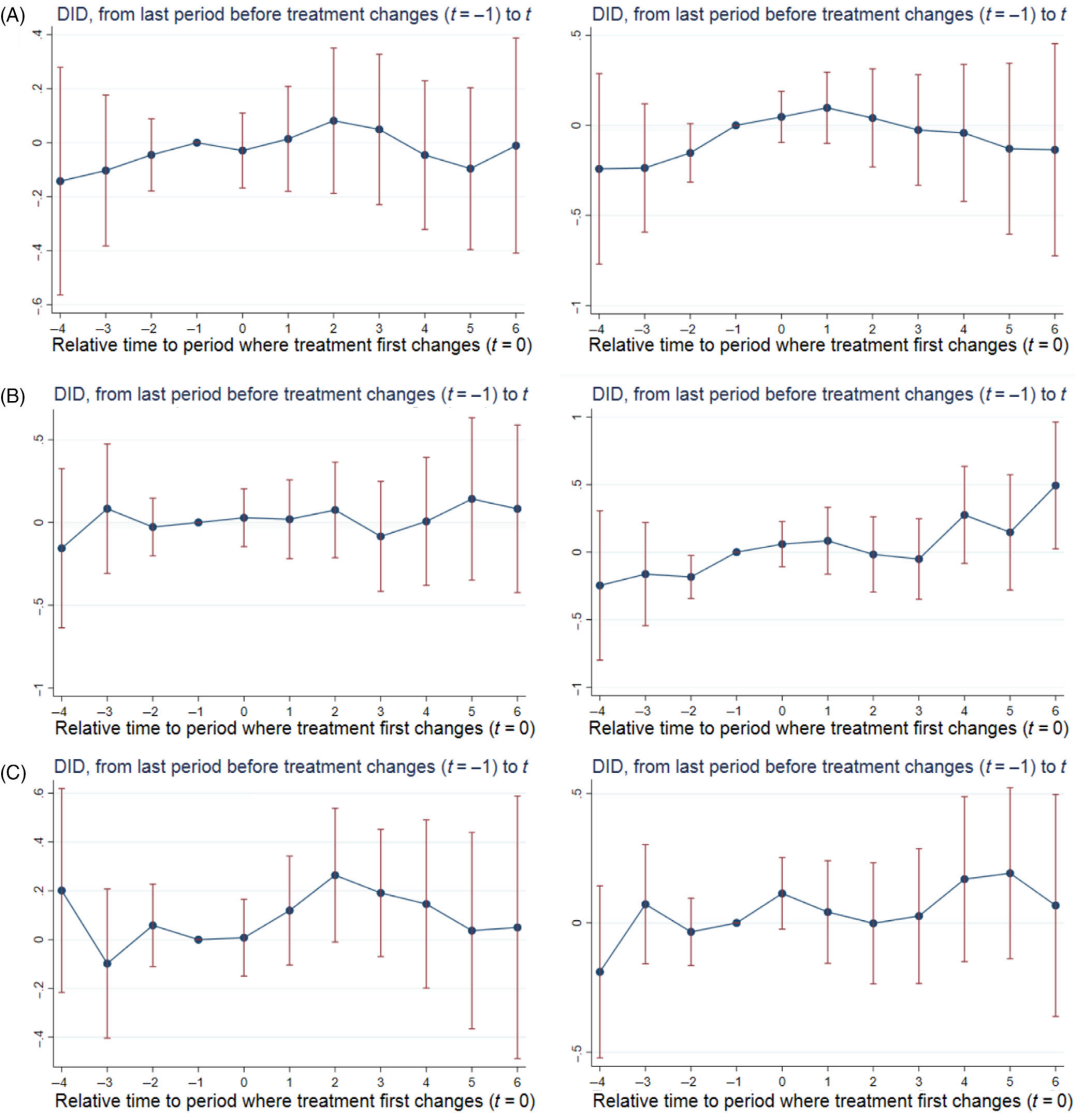
Effect of cross‐market M&A on acquirers' quality. (A) Heart Failure. (B) Heart Attack. (C) Pneumonia. Standard errors were estimated using 100 bootstrap replications clustered at the hospital level. The regressions underlying these event study plots included hospital and year fixed effects as well as time‐varying hospital‐ and county‐level control variables. The hospital‐level control variables included number of beds, indicator variables for the hospital's for‐profit, government, or teaching hospital status, and the hospital's share of inpatient days from Medicare and Medicaid enrollees as well as its number of technologies. The county‐level control variables included number of hospitals, uninsured rate, median household income, population, and unemployment rate. DID, difference‐in‐differences; M&A, mergers and acquisitions; *t*, time since treatment first changes.

Figure 4 shows the quality effect of cross‐market hospital mergers. Panel A shows the event studies when heart failure mortality and heart failure readmission rate are the dependent variables. In both cases there is no noticeable pre‐trend and none of the post‐treatment coefficients are statistically different from zero. Panel B likewise shows minimal to no impact of cross‐market mergers on acquirer quality when heart attack mortality and readmission rate are the dependent variables. None of the post‐treatment coefficients are statistically different from zero for heart attack mortality. For the heart attack readmission rate event study, the t=6 coefficient is positive and statistically significant (0.494;p=0.039) which suggests cross‐market mergers reduce acquirer quality by increasing the heart attack readmission rate, however, there was a pre‐treatment trend in this case so this result is ambiguous. Panel C shows the event studies for pneumonia mortality and readmission rate. Just as in Panel A, both plots show the post‐treatment coefficients all being close to zero and not statistically significant. Overall, our results point to cross‐market mergers having no impact on acquirer quality.

## 5 DISCUSSION

This article contributes to the small, but growing, literature that analyzes cross‐market hospital mergers and acquisitions and examines whether they can lead to price increases and harm competition.^5^, ^6^ Similar to those studies, we find that cross‐market hospital acquisitions are associated with acquirer price increases of 12.9% as compared with controls, 6 years following the merger or acquisition. Our results suggest there is a time delay of a few years following successful completion of the merger before price effects emerge, which may be due to existing contracts with insurers or a desire to not immediately increase prices for other reasons.

In addition, we found larger price effects when the acquirer had lower market share than the target, although significant price increases were still found when the opposite was true. This finding makes intuitive sense, as acquirers with lower market share have more to gain from acquiring an entity with market power. We also found that the price effects following cross‐market acquisitions existed for both within‐state transactions and out‐of‐state transactions, but price effects of within‐state transactions emerged earlier post‐transaction. In contrast to Dafny et al.^6^ our findings suggest that cross‐market price effects extend across state lines, consistent with the theory that when common customers, such as the big 5 national insurers,^20^ negotiate with multi‐hospital systems that cross state lines, they can be subject to their market power.

In addition to being the first study to use healthcare claims data to find that cross‐market acquisitions result in price increases, our novel contribution to the literature is that we have disentangled some of the price effects to provide guidance on the characteristics of acquiring hospital systems and cross‐market mergers that are likely to have the greatest price effects over time. Our analysis of health systems that engaged in 4 or more cross‐market acquisitions between 2011 and 2017 (approximately 45% of treated hospitals) revealed that those serial cross‐market acquirers had 16.3% higher prices than controls 6 years after the acquisition. In contrast, health systems that acquired three or fewer cross‐market entities during the study period showed some signs of price effects at year 4 but they proved transitory over time. We also analyzed the impact of cross‐market hospital acquisitions on six quality measures and found no significant quality effects, suggesting that the price effects do not arise from post‐transaction improvements in quality of care.

Our study has several limitations. First, the claims we used to calculate prices came from only three insurers – UnitedHealth, Aetna, and Humana. While these three insurers are large, national players, they account for only about a third of employer‐sponsored health insurance enrollment in the United States. We expect the prices these three insurers receive to be correlated with those of other insurers, but to the extent that they are not, our price results could be biased (in either direction). It seems unlikely that quality would differ by insurer within the same hospital, but there could also be some bias in our quality estimates if the in‐network hospitals for these three insurers differed from the in‐network hospitals of other insurers. Second, we do not answer the distance gradient question of how the price effect changes as the distance between cross‐market hospital targets and acquirers grows. We use a 50‐mile threshold to define cross‐market, but we are not able to comment on whether a 100‐mile cross‐market transaction has a larger price effect than a 300‐mile cross‐market transaction. Third, by reducing to a single hospital price we cannot provide detail on how changes in prices may vary heterogeneously for each specific DRG or service line. Fourth, we are unable to pinpoint a primary cross‐market mechanism that is at work here. By focusing on acquirers' prices, we think it is unlikely that change‐in‐control or quality improvements explain the observed price increases, but whether tying, common customers, or multimarket contact is largely responsible remains unclear. These limitations are important for policymakers and antitrust regulators to consider in light of our findings. Future research that identifies the mechanism (or degree to which multiple mechanisms contribute) will be particularly useful in terms of guiding policymakers and antitrust regulators.

## 6 CONCLUSION

Our findings provide additional empirical evidence of the potential price effects arising from healthcare system consolidation broadly and cross‐market hospital acquisitions specifically. Our study also provides key guidance for antitrust enforcers and policymakers on the characteristics of health systems and acquisitions that are most likely to contribute to enduring price effects. More antitrust scrutiny of cross‐market mergers – particularly those of serial acquirers – appears prudent given the current state of highly concentrated hospital markets in the United States.

## Supporting information

Appendix
