# Supplementary material for: New evidence on the impacts of cross‐market hospital mergers on commercial prices and measures of quality
Source: Health Serv Res. 2024 Apr 23;60(1):e14291. doi: 10.1111/1475-6773.14291 (PMC11782062; doi:10.1111/1475-6773.14291)
Supplement: Supplementary file 1 — Appendix [file HESR-60-0-s001.pdf]

7. de Chaisemartin C, D'Haultfoeuille X. Difference-in-differences estimators of intertemporal treatment effects. *Rev Econ Stat*. 2023. Forthcoming. https://doi.org/10.1162/rest_a_01414
8. King JS, Montague AD, Arnold DR, Greaney TL. Antitrust's healthcare conundrum: cross-market mergers and the rise of system power. *Hastings Law J*. 2023;74(4):1057-1120.
9. Whinston MD. Tying, foreclosure, and exclusion. *Am Econ Rev*. 1990; 80(4):837-859.
10. Nalebuff BJ. *Bundling as a Way to Leverage Monopoly*. Social Science Research Network; 2004. https://papers.ssrn.com/abstract=586648
11. Schmalensee R. Monopolistic two-part pricing arrangements. *Bell J Econ*. 1981;12(2):445-466. doi:10.2307/3003566
12. Bernheim BD, Whinston MD. Multimarket contact and collusive behavior. *Rand J Econ*. 1990;21(1):1-26. doi:10.2307/2555490
13. Schmitt M. Multimarket contact in the hospital industry. *Am Econ J Econ Policy*. 2018;10(3):361-387. doi:10.1257/pol.20170001
14. Lin H, McCarthy IM. Multimarket contact in health insurance: evidence from Medicare advantage. *J Ind Econ*. 2023;71(1):212-255. doi: 10.1111/joie.12318
15. Cooper Z, Doyle Jr Joseph J, Graves JA, Gruber J. Do higher-priced hospitals deliver higher-quality care? 2022. doi:10.3386/w29809
16. Cooper Z, Craig SV, Gaynor M, Van Reenen J. The price ain't right? Hospital prices and health spending on the privately insured. *Q J Econ*. 2019;134(1):51-107.
17. Scheffler RM, Arnold DR. Insurer market power lowers prices in numerous concentrated provider markets. *Health Aff (Millwood)*. 2017;36(9):1539-1546.
18. Beaulieu ND, Dafny LS, Landon BE, Dalton JB, Kuye I, McWilliams JM. Changes in quality of care after hospital mergers and acquisitions. *N Engl J Med*. 2020;382(1):51-59.
19. Roth J, Sant'Anna PHC, Bilinski A, Poe J. What's trending in difference-in-differences? A synthesis of the recent econometrics literature. *J Econom*. 2023;235(2):2218-2244. doi:10.1016/j.jeconom.2023.03.008
20. Schoen C, Collins SR. The big five health insurers' membership and revenue trends: implications for public policy. *Health Aff (Millwood)*. 2017;36(12):2185-2194.

How to cite this article: Arnold DR, King JS, Fulton BD, et al. New evidence on the impacts of cross-market hospital mergers on commercial prices and measures of quality. *Health Serv Res*. 2025;60(1):e14291. doi:10.1111/1475-6773.14291

APPENDIX A

ECONOMETRIC MODEL TECHNICAL DETAILS AND IDENTIFYING ASSUMPTIONS

De Chaisemartin and D'Haultfoeuille (Forthcoming)* (hereafter, dCDH) take the perspective of a social planner seeking to conduct a cost–benefit analysis comparing groups' (hospitals') actual treatments to the counterfactual "status-quo" scenario where every group would have kept the same treatment (i.e., not acquired) as in period 1. In our context, the planner wants to know if the cross-market mergers that took place over the entire duration of the study period led to prices and quality to be higher or lower. As the planner wants to compare groups' actual treatments D to the status-quo treatments, the dCDH parameters of interest and all of their analysis are conditional on D.

Consider the case where treatment is not binary, but ordered and discrete: $D \in \{1,\dots,\underline{d}\}$ for $\underline{d} \geq 1$. For every g, let $T_g = F_{g'} - 1$ denote the last period where there is still a group with the same treatment as g's in period one and whose treatment has not changed since the start of the panel. For any g such that $F_g \leq T_g$, and for any $l \in \{0,\dots,T_g - F_g\}$, let $\delta_{g,l} = E\left(Y_{g,F_g+l} - Y_{g,F_g+l}(D_{g,1},\dots,D_{g,1})\right)$ be the expected difference between group g's actual outcome at $F_g + l$ and the counterfactual "status quo" outcome it would have obtained if its treatment had remained equal to its period one value from period one to $F_g + l$. dCDH consider designs where (1) groups' treatments are always either weakly higher or always weakly lower than their period one treatments and (2) there is at least one group g experiencing a treatment increase (decrease) at a time period where there is at least another group g' with the same period one treatment as g whose treatment has not changed since the start of the panel. (1) is satisfied in our context because each additional year that a treated hospital acquires a cross-market hospital adds 1 to its treatment dose (i.e., treatment never decreases) and (2) is satisfied because our control group of hospitals maintain a treatment dose of 0 throughout the study period. de dCDH show that if (1) and (2) are true then their parameters of interest are well-defined and can be unbiasedly estimated.

The identifying assumptions outlined in dCDH for ordered and discrete treatments are (1) treatment does not vary within (g,t) cells, (2) no anticipation, and (3) for every hospital the expectation of the never-treated outcome follows the same evolution. Assumption (3) is a generalization of the standard parallel trends assumption in difference-in-differences models and we test for parallel trends using the placebo estimator in dCDH. Assumption 1 holds by construction in our setting and we do not observe any evidence of assumption 2 being violated.

For all (g,t), let $N_t^g = \sum_{g':D_{g',1}=D_{g,1},F_{g'}>t} N_{g',t}$ denote the number of observations at period t in groups g' with the same period one treatment as g, and that kept the same treatment from period 1 to t. Under the three assumptions in the prior paragraph, de Chaisemartin and D'Haultfoeuille (2022) show $DID_{g,l}$ is an unbiased estimator of $\delta_{g,l}$ if

$$DID_{g,l} = Y_{g,F_g+l} - Y_{g,F_g-1} - \sum_{g':D_{g',1}=D_{g,1},F_{g'}>F_g+l} \frac{N_{g',F_g+l}}{N^g_{F_g+l}} \left(Y_{g',F_g+l} - Y_{g',F_g-1}\right).$$

$DID_{g,l}$ compares the $F_g - 1$-to-$F_g + l$ outcome evolution, in group g and in groups with g's period one treatment to period 1 to $F_g + 1$. Aggregating the $DID_{g,l}$ estimators into a DID_l estimator allows for the creation of an event study graph that has the distance to the first treatment change on the x-axis, the DID_l estimators on the y-axis to the right of zero, and placebo estimators on the y-axis. The resulting event study graph is useful to (1) test the parallel trends assumption and (2) provide reduced-form evidence of whether increasing the treatment for $l+1$ periods increases or decrease the outcome on average.

*De Chaisemartin C, D'Haultfoeuille X. Difference-in-Differences Estimators of Intertemporal Treatment Effects. *Rev Econ Stat*. Published online Forthcoming.

TABLE A1 Number of years that a treated hospital's system acquired a hospital more than 50 miles away between 2011 and 2017.

Number of years	Number of treated hospitals	Number of treated hospitals' systems
1	32	17
2	43	9
3	43	9
4	28	6
5	16	3
6	30	2
7	22	1
Total	214	47

Note: Treatment hospitals included hospitals (or hospitals within systems) that met the following criteria: (1) hospitals that made an acquisition from 2009 to 2017 of a hospital (or system) that was further than 50 miles away, with the first acquisition occurring from 2011 to 2015; and (2) hospitals that were never a target of an acquisition from 2009 to 2017. For example, a treated hospital whose system acquired one or more hospitals that were more than 50 miles away from it in 2011, 2013, and 2016 (3 years between 2011 and 2017) was included in the row in which the "Number of Years" column equaled 3. Control hospitals were never part of merger activity (either as a target or acquirer) from 2009 to 2017.

TABLE A2 Price event study regression coefficient estimates underlying Figure 1.

	(1)
	ln (price)
$t=-4$	0.020
	(0.025)
$t=-3$	0.010
	(0.010)
$t=-2$	0.012
	(0.014)
$t=0$	0.013
	(0.017)
$t=1$	0.008
	(0.034)
$t=2$	0.029
	(0.023)
$t=3$	0.061***
	(0.020)
$t=4$	0.072**
	(0.032)
$t=5$	0.054**
	(0.026)
$t=6$	0.121**
	(0.059)
Observations	10,521
Time-varying control variables	Yes
Fixed effects	Hospital, Year

Note: Standard errors are in parentheses and were estimated using 100 bootstrap replications clustered at the hospital level. The coefficients estimates are depicted in Figure 1 in the main text; $t=-1$ is the omitted reference period. The time-varying control variables are described in the data section of the main text. ***$p < 0.01$; **$p < 0.05$; *$p < 0.1$.

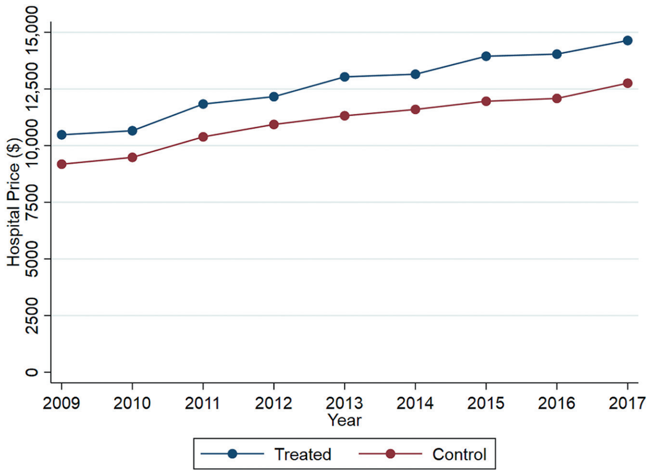

FIGURE A1 Unadjusted price trends for treated and control hospitals. Average hospital prices across the 214 treated hospitals and 955 control hospitals in our analytic sample. By construction the treated group includes only hospitals that were first treated during the 2011–2015 window of our study period (2009–2017). The breakdown by treatment year for the 214 treated hospitals is 80 in 2011, 31 in 2012, 49 in 2013, 37 in 2014, and 17 in 2015.

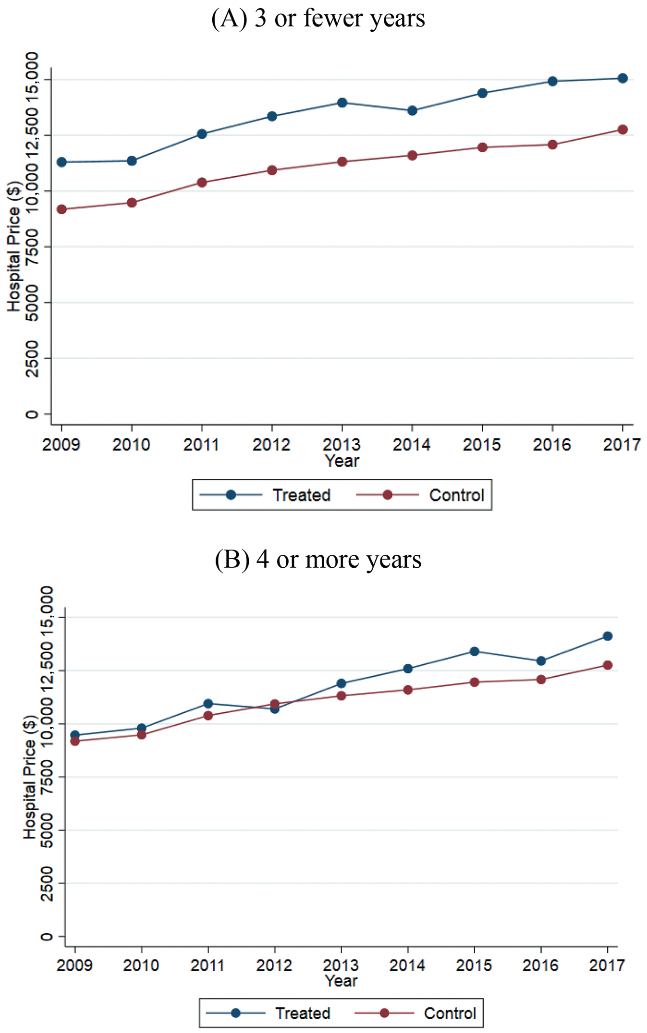

FIGURE A2 Unadjusted price trends for treated and control hospitals by number of years from 2011 to 2017 that treated hospitals' systems acquired hospitals across markets. (A) 3 or fewer years. (B) 4 or more years. Average hospital prices across the 214 treated hospitals and 955 control hospitals in our baseline sample. Panel A shows the average price across the 118 treated hospitals that were part of systems that acquired cross-market hospitals in 3 or fewer years from 2011 to 2017. Panel B shows the average price across the 96 treated hospitals that were part of systems that acquired cross-market hospitals in 4 or more years from 2011 to 2017. The control group line is the same in Panels A and B and is the same control group line plotted in Figure A1.

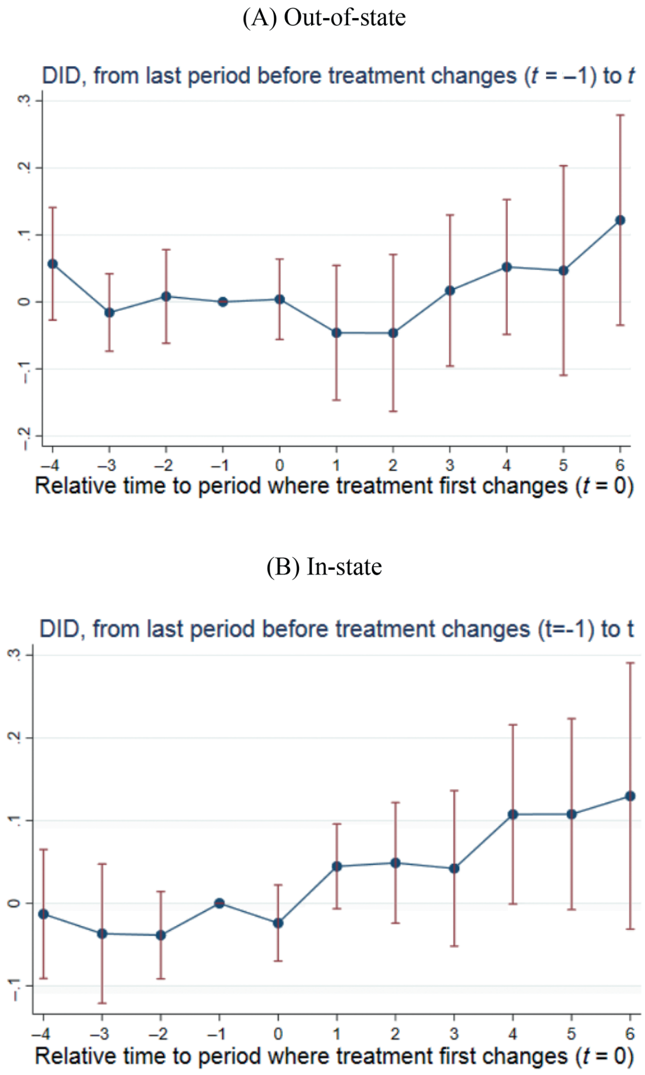

FIGURE A3 Price event study results – out-of-state vs. in-state cross-market mergers. (A) Out-of-state. (B) In-state. Standard errors were estimated using 100 bootstrap replications clustered at the hospital level. Panel A includes the 68 treated hospitals that were only out-of-state cross-market acquirers between 2011 and 2017. Panel A includes the 60 treated hospitals that were only in-state cross-market acquirers between 2011 and 2017. The remaining 86 (=214–68-60) treated hospitals were excluded from this analysis because they were part of systems that made both in-state and out-of-state cross-market acquisitions during the study period. The regressions underlying these event study plots included hospital and year fixed effects as well as the time-varying hospital- and county-level control variables described in the data section of the main text.
